# ZFPM2-AS1 transcriptionally mediated by STAT1 regulates thyroid cancer cell growth, migration and invasion via miR-515-5p/TUSC3

**DOI:** 10.7150/jca.51437

**Published:** 2021-04-17

**Authors:** Ruizhen Ren, Yuanna Du, Xing Niu, Rukun Zang

**Affiliations:** 1 Department of Endocrinology, Yantai Yuhuangding Hospital Affiliated to Qingdao University, Yantai 264000, Shandong, China.; 2 Department of Radiotherapy, Yantai Yuhuangding Hospital Affiliated to Qingdao University, Yantai 264000, Shandong, China.; 3 Department of Second Clinical College, Shengjing Hospital of China Medical University, Shenyang 110004, Liaoning, China.

**Keywords:** thyroid cancer, ZFPM2-AS1, TUSC3, miR-515-5p, transcription factor.

## Abstract

**Objective:** Our purpose was to study the roles and molecular mechanisms of long non-coding RNA (lncRNA) ZFPM2 Antisense RNA 1 (ZFPM2-AS1) in thyroid cancer.

**Methods:** Firstly, the expression of ZFPM2-AS1, miR-515-5p and TUSC3 was detected in thyroid cancer tissues and cells. Secondary, their biological functions (proliferation, apoptosis, migration and invasion) were analyzed by a serious of functional experiments including cell counting kit-8 (CCK-8), clone formation, 5-Ethynyl-2'-deoxyuridine (EdU), enzyme-linked immunosorbent assay (ELISA), wound healing and Transwell assays. Thirdly, the mechanisms of STAT1/ZFPM2-AS1 and ZFPM2-AS1/miR-515-5p/TUSC were validated using chromatin immunoprecipitation (CHIP), pull-down and luciferase reporter assays.

**Results:** ZFPM2-AS1 and TUSC were both highly expressed and miR-515-5p was down-regulated in thyroid cancer tissues as well as cells. Their knockdown weakened thyroid cancer cell growth, migration, and invasion. ZFPM2-AS1 was mainly distributed in the nucleus and cytoplasm of thyroid cancer cells. Mechanistically, up-regulation of ZFPM2-AS1 was induced by transcription factor STAT1 in line with CHIP and luciferase reporter assays. Furthermore, as a sponge of miR-515-5p, ZFPM2-AS1 decreased the ability of miR-515-5p to inhibit TUSC3 expression by pull-down, luciferase reporter and gain-and-loss assays, thereby promoting malignant progression of thyroid cancer.

**Conclusion:** ZFPM2-AS1 acted as an oncogene in thyroid cancer, which was transcriptionally mediated by STAT1. Furthermore, ZFPM2-AS1 weakened the inhibitory effect of miR-515-5p on TUSC3. Thus, ZFPM2-AS1 could be an underlying biomarker for thyroid cancer.

## Introduction

Thyroid cancer is the most frequently diagnosed malignancy in the endocrine system 1. The current treatment for thyroid cancer is relatively single 2. Despite most patients can achieve optimistic clinical outcomes with only surgery, there are still a lack of other effective treatments for some patients with strong invasion and metastasis, which seriously affects the life quality of patients, and imposes a double burden on the economy and spirit 3. Thyroid cancer is a multifactorial disease affected by environment and genes. The currently discovered genes related to thyroid cancer are not yet sufficient to fully explain the pathophysiological process of thyroid cancer, and no breakthrough has been made in the clinical diagnosis and treatment of thyroid cancer. Therefore, the discovery of novel diagnostic markers of thyroid cancer is urgently required 3. LncRNA (>200bp), a non-coding transcript, possesses little protein coding potential 4. Increasing evidences suggest that lncRNAs may regulate the expression of targeted mRNA s via sponging miRNAs, thereby controlling the key processes of cells, and participating in tumor pathogenesis, including thyroid cancer 5. LncRNAs could be promising diagnostic and therapeutic targets for thyroid cancer 6. However, despite the changes in lncRNA in thyroid cancer have been widely recognized, most of functions and their molecular mechanisms of thyroid cancer-related lncRNAs have not yet been determined.

ZFPM2-AS1 is located at 8q23.1. Recently, ZFPM2-AS1 has been found to mediate the progression of several cancers. It can promote gastric cancer development via stabilizing MIF 7. ZFPM2-AS1 accelerates migration and proliferation of renal cell carcinoma cells via targeting miR-137 8. ZFPM2-AS1 speeds up lung adenocarcinoma progression by miR-511-3p/AFF4 9, UPF1-ZFPM2 10 and miR-18b-5p/VMA21 pathways 11. ZFPM2-AS1 promotes the growth of esophageal squamous cell carcinoma cells by up-regulation of TRAF4 and NF-κB 12. Silencing ZFPM2-AS1 weakens retinoblastoma progression through mediating miR-515/HOXA1 axis 13. Nevertheless, its roles in thyroid cancer are still undiscovered.

Herein, we determined ZFPM2-AS1 expression and its diagnostic value in thyroid cancer. We studied the effects of ZFPM2-AS1 expression changes on biological behaviors of thyroid cancer cells. Mechanistically, ZFPM2-AS1 was transcriptionally regulated by STAT1 and bound to miR-515-5p through competitive competition with TUSC3 in thyroid cancer cells.

## Materials and methods

### Patients and samples

90 cases of thyroid cancer patients who received surgical treatment were recruited in Yantai Yuhuangding Hospital Affiliated to Qingdao University between March, 2017 and March, 2019. Thyroid cancer was confirmed by postoperative pathological examination. These patients did not undergo ^131^I internal radiation therapy, thyrotropin suppression therapy or conventional radiation therapy before enrollment. Thyroid tumor and adjacent normal thyroid tissues were collected during the operation, which were placed in liquid nitrogen right away and quickly frozen, and then transferred to a -80 °C for storage. This project gained the approval of the ethics committee of Yantai Yuhuangding Hospital Affiliated to Qingdao University (2017019). The study strictly followed the ethical principles of the Declaration of Helsinki. All participants provided written informed consents.

### Cell culture

Human thyroid normal cells Nthy-ori3-1 and human thyroid cancer cells (K1, SW579 and 8505C) were purchased from Shanghai Institute of Cell Research, Chinese Academy of Sciences (China). All cells were grown in DMEM medium (Gibico, Grand Island, USA) plus 10% FBS in a 5% CO_2_ environment at 37 °C.

### Quantitative real-time PCR (qRT-PCR)

Total RNA was extracted from tissues or cells utilizing total RNA extraction reagent (Promega, Madison, Wisconsin, USA), which was then reverse transcribed into cDNA via reverse transcription reaction kit Takara, Japan). qRT-PCR was presented via ABI7500 quantitative PCR system (Applied Biosystems, Warring ton, UK) and SYBR Green PCR Kit (Takara). Relative expression levels were determined with the 2^-ΔΔCt^ method 8. Primers were designed and synthesized by Sangon Biotech (Shanghai, China). Primer sequences were as follows: ZFPM2-AS1: 5'-CAATGGGACTAAGCCAGGCA-3' (forward), 5'-GGGCTCCACCAACAACCATA-3' (reverse); miR-515-5p: 5'-TTCTCCAAAAGAAAGCACTTTCTG-3'; TUSC3: 5'-GGCTCAGTTTGTGGCAGAATC-3' (forward), 5'-CATCGCCTTTCGAAGTTGCT-3' (reverse); GAPDH: 5'-GTCAACGGATTTGGTCTGTATT-3' (forward), 5'-AGTCTTCTGGGTGGCAGTGAT-3' (reverse).

### Transfection

Cells were seeded onto a 12-well plate. si-STAT1, si-ZFPM2-AS1, si-TUSC3, pcDNA3.1-STAT1, pcDNA3.1-ZFPM2-AS1 miR-515-5p mimics/inhibitor, pcDNA3.1-TUSC3 and their controls were transfected into cells via Lipofectamine 3000 (Invitrogen, California, USA) for 48 h, which were purchased by Shanghai GenePharma Co., Ltd (China).

### Chromatin immunoprecipitation (CHIP) assay

CHIP was presented using CHIP kit (Beyotime, Beijing, China). The cells were cultured in 10 cm petri dishes, which were incubated with 270 µl of 37% formaldehyde at 37 °C for 10 min and 1.1 ml 1M glycine at room temperature for 5 min. The cells were lysed on the ice for 10 min. Ultrasound was used to disrupt the cells, making the DNA break at 200-1000 bp. Subsequently, the sonicated samples were centrifuged at 12,000 g at 4 °C for 5 min, and the supernatant was collected. 20 µl samples were used as Input group. 70 µl Protein A+G Agarose/Salmon Sperm DNA was added to the remaining samples and mixed at 4 °C for 30 min. After that, the samples were immunoprecipitated with STAT1 (1:100) and IgG antibodies. IgG antibody was set as a negative control. 60 µl Protein A+G Agarose/Salmon Sperm DNA was added to samples, and shook gently at 60 °C for 4 min to identify the target protein bound by the primary antibody. PCR was presented to amplify the sequence of the target gene.

### Dual luciferase report

The cells were transfected with a luciferase plasmid plus the target sequence and a Renilla luciferase plasmid for 48 h. The dual luciferase reporter gene activity test was presented through the Dual-Glo® Luciferase Assay System kit (Promega, Madison, Wisconsin, USA). Firefly and renilla luciferase activity values were determined using Tecan® infinite M200 multi-function microplate reader (Tecan, Shanghai, China), thereby calculating the relative luciferase activity.

### Cell counting kit-8 (CCK-8) assay

Transfected cells were seeded in 96-well culture plates (3×10^3^/well) and cultured in a 35% CO_2_ incubator at 37 °C for 6-8 h. At 24 h, 48 h, 72 h and 96 h after transfection, 10 μl of CCK-8 solution (Dojindo, Japan) was added to each well and incubated for 1 h. The microplate reader (Molecular Device, Silicon Valley, USA) was utilized to monitor the optical density of each well at 450 nm wavelength.

### Clone formation experiment

Transfected cells were planted into 6-well plates (1000 cells/well). The medium was changed every 5 days. After 2 weeks, the samples were fixed by 4% paraformaldehyde for 30 min and stained by 0.5% crystal violet for 15 min. Colony numbers were then counted under a light microscope (Olympus, Japan).

### 5-Ethynyl-2'-deoxyuridine (EdU)

EdU was presented via EdU Proliferation Detection Kit (RIBOBIO, Guangzhou, China). Briefly, after being incubated with 100 μL 50 μM EdU medium for 2 h, the cells were washed with PBS to elute EdU that did not penetrate DNA. The cells were then fixed with 50 μL cell fixation solution at room temperature for 30 min and were incubated with 50 μL 2 mg/mL glycine for 5 min. After washing, the cells were incubated with 100 μL 1×Apollo® staining reaction solution and 100 μL 1×Hoechst 33342 reaction solution at room temperature in the dark for 30 min, respectively. Results were immediately observed.

### ELISA assay

In line with the manufacturer's instructions, cleaved Caspase-3 and Caspase-9 levels were detected utilizing human cleaved Caspase-3 ELISA kit (ab220655) and human Caspase-9 ELISA kit (ab119508).

### Wound healing experiment

A ruler was used to draw parallel lines on the back of a 6-well plate. Transfected cells were seeded on the plate. After the cells were starved with serum-free medium for several hours, a horizontal line was scratched on the back by a pipette tip. After washing off the scraped cells with PBS, the plate was cultured in an incubator. After 24 h, the results were investigated.

### Transwell assay

Matrigel (BD, New Jersey, USA) was evenly spread on the bottom surface of the transwell chamber (Corning, Shanghai, China). 500 μl medium containing 10% FBS was added in the lower chamber. 2×10^4^ cells were plated on the upper chamber. After discarding the culture medium, the cells were stained with crystal violet for 5 min. Then, the cells that had not passed through the upper chamber were wiped off. Following washing with PBS and drying, images were taken under a microscope (Olympus, Japan).

### Western blot

Tissues or cells were lysed utilizing RIPA lysate (Beyotime) on the ice. The sample was centrifuged at 12,000 g for 20 min at 4 °C. The supernatant was collected and stored at -80 °C. Protein concentration was examined via BCA protein concentration detection kit (Beyotime). Protein sample was separated via SDS-PAGE and transferred into PVDF membrane (Bio-Rad, Hercules, CA). 5% non-fat soluble milk liquid was utilized to block the membrane for 1 h. Afterwards, the membrane was incubated with primary antibodies including E-cadherin (1:1000, ab133597, abcam, Cambridge Science Park, UK), N-cadherin (1:1000, ab207608), Vimentin (1:1000, ab92547) and GAPDH (1:1000, ab9485) at 4 °C overnight, followed by goat anti-rabbit IgG H&L (HRP; 1:5000, ab205718, abcam) at room temperature for 1 h. Protein blots were visualized utilizing ECL chemiluminescence reagent (Pierce, Rockford, USA). The grayscale value was measured using the ImageJ software.

### Subcellular fractionation location

Nuclear/Cytosol Fractionation Kit (Beyotime) was utilized to separate nuclear and cytosol of RNA. U6 and GAPDH were utilized as nuclear and cytosolic controls, respectively.

### Pull-down assay

This assay was presented using magnetic RNA protein pull-down kit (Pierce, USA). 50 pmol biotin ZFPM2-AS1 (RiboBio, China) was treated with 50 μl streptavidin-agarose beads at 4 °C for 1 h. Then, the RNA-bound magnetic beads were added to the lysate, followed by qRT-PCR.

### Statistical analysis

SPSS 17.0 software was utilized for statistical analyses. Experimental results were expressed as mean ± standard deviation. Student's t test was used for comparison between two groups, and one-way ANOVA was presented for multiple comparisons. Bioinformatics analysis was presented using R language version 3.5.3. p<0.05 was considered statistically significant.

## Results

### Expression and clinical features of ZFPM2-AS1 in thyroid cancer

**Figure 1:**
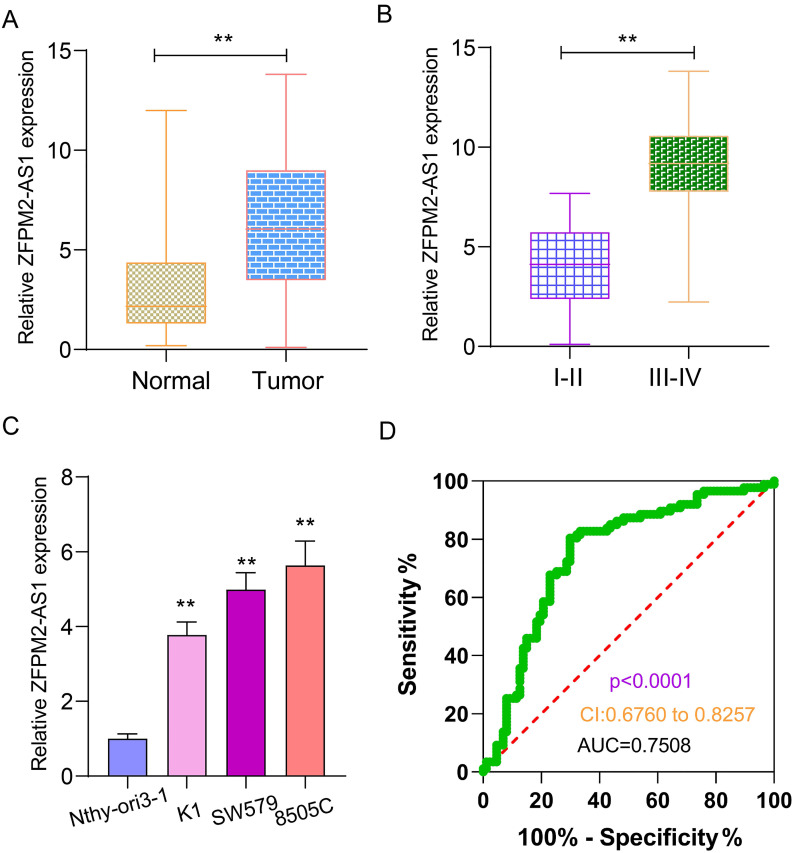
** Expression and clinical features of ZFPM2-AS1 in thyroid cancer. (A)** High ZFPM2-AS1 expression was detected in thyroid cancer tissues via qRT-PCR. **(B)** The difference in ZFPM2-AS1 expression between I-II and III-IV thyroid cancer patients. **(C)** qRT-PCR was utilized to monitor ZFPM2-AS1 expression in thyroid cancer and normal cells. (D) ROC was depicted to determine the efficiency of ZFPM2-AS1 expression as a diagnosed marker of thyroid cancer. **p<0.01.

qRT-PCR examined ZFPM2-AS1 expression in 90 pairs of thyroid cancer and normal tissues. As shown in Figure 1A, ZFPM2-AS1 expression was distinctly higher in tumor tissues in comparison to normal tissues. Its high expression was significantly associated with clinical stage (Figure 1B). Patients with III-IV usually had higher ZFPM2-AS1 expression in comparison of those with I-II. ZFPM2-AS1 expression was also detected in human thyroid normal Nthy-ori3-1 cells and human thyroid cancer cells (K1, SW579 and 8505C). qRT-PCR results showed that there was a higher ZFPM2-AS1 expression in thyroid cancer cells than normal cells (Figure 1C). SW579 and 8505C cells had a relatively high basic expression of ZFPM2-AS1, therefore, these two cell lines were employed to study the function of ZFPM2-AS1. The ROC results demonstrated that ZFPM2-AS1 expression could be a sensitive diagnostic biomarker for thyroid cancer (Figure 1D).

### ZFPM2-AS1 is transcriptionally regulated by STAT1 in thyroid cancer

**Figure 2:**
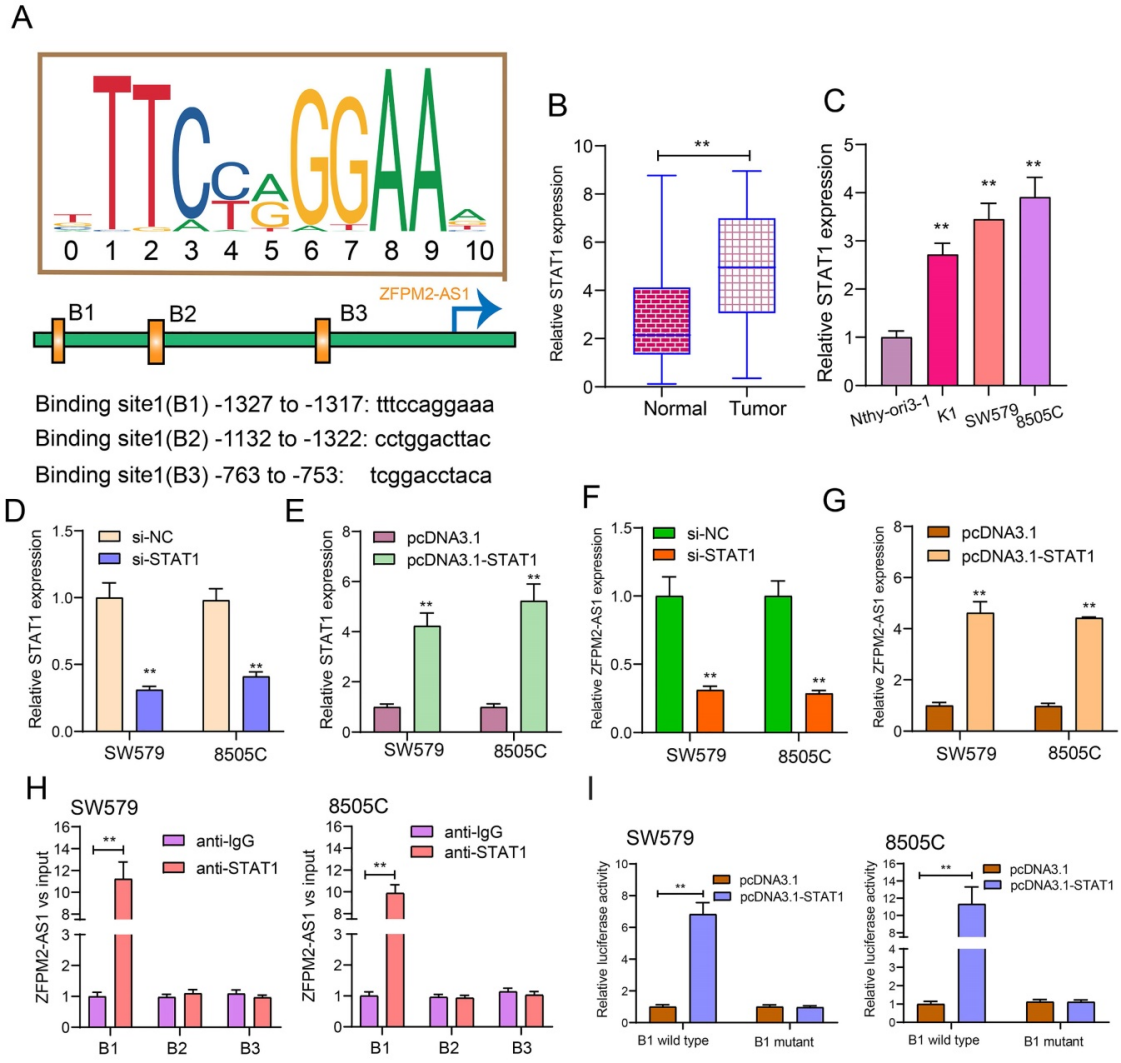
** ZFPM2-AS1 is transcriptionally regulated by STAT1 in thyroid cancer. (A)** Schematic diagram showing the binding sites in the promoter region of ZFPM2-AS1 for STAT1. **(B, C)** qRT-PCR examining STAT1 expression in thyroid cancer tissues as well as cells. **(D, E)** qRT-PCR verifying the transfected efficiencies of si-STAT1 and pcDNA3.1-STAT1 in thyroid cancer SW579 and 8505C cells. **(F, G)** ZFPM2-AS1 expression was tested in SW579 and 8505C cells with si-STAT1 and pcDNA3.1-STAT1. **(H)** ChIP assay and **(I)** Dual luciferase reporter assay validating the binding sites of STAT1 and ZFPM2-AS1 promoter region. **p<0.01.

After prediction using the JASPAR database (http://jaspar.genereg.net/), there were three binding sites in the promoter region of ZFPM2-AS1 for STAT1 (Figure 2A). Our data showed that STAT1 expression was raised both in thyroid cancer tissues (Figure 2B) and cells (Figure 2C). Then, we respectively silenced (Figure 2D) and overexpressed (Figure 2E) STAT1 in two thyroid cancer cells. After its knockdown, ZFPM2-AS1 expression was markedly decreased in thyroid cancer cells (Figure 2F). Conversely, there was a higher ZFPM2-AS1 expression following transfection with pcDNA3.1-STAT1 (Figure 2G). The ChIP analysis suggested that STAT1 only bound to the binding site 1 (B1; -1327 to -1317bp upstream of TSS) of ZFPM2-AS1 promoter region, not B2 or B3 (Figure 2H), which was confirmed by the luciferase report assay (Figure 2I). Compared to mutant B1, STAT1 was distinctly bound to wild B1. Above results demonstrated that ZFPM2-AS1 could be transcriptionally regulated by STAT1 in thyroid cancer.

### ZFPM2-AS1 knockdown weakens proliferation and facilitates apoptosis for thyroid cancer cells

**Figure 3:**
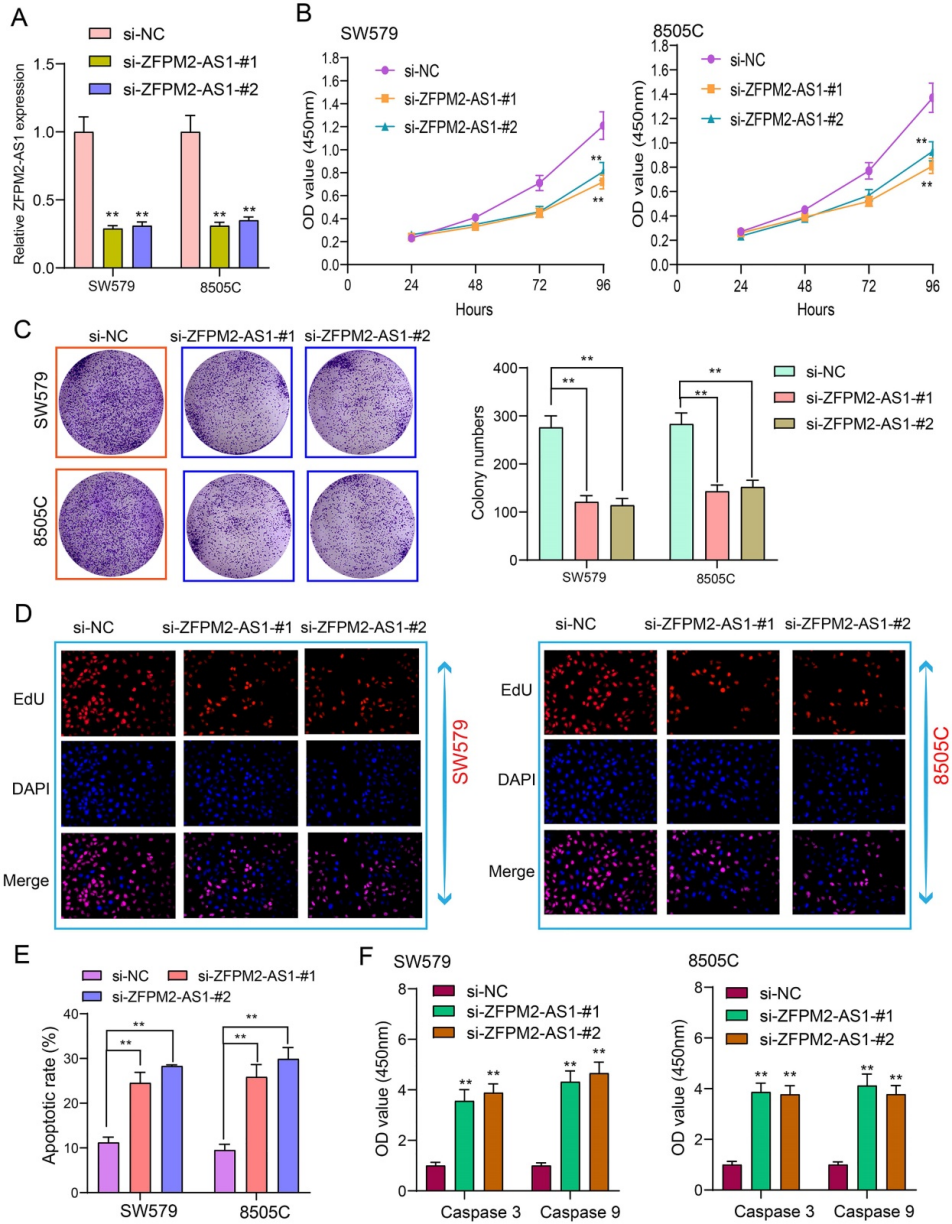
** ZFPM2-AS1 knockdown weakens proliferation and facilitates apoptosis for thyroid cancer cells. (A)** siRNAs were used to interfere with ZFPM2-AS1 expression in SW579 and 8505C cells. **(B)** CCK-8 experiment detected that the viability of SW579 and 8505C cells with si-ZFPM2-AS1 was suppressed. **(C)** Clone formation experiment showed that the number of clone formations was weakened in SW579 and 8505C cells with si-ZFPM2-AS1. **(D, E)** EdU experiment detecting the changes in cell proliferation after transfection of si-ZFPM2-AS1 as well as its corresponding negative controls. Scale bar: 20 μm. Magnification: 200×. **(F)** ELISA showed lower Caspase 3 and 9 levels in cells with si-ZFPM2-AS1. **p<0.01.

Tumor cells have the characteristics of monoclonal growth. Inhibiting the proliferation of tumor cells has a long-term significance for thyroid cancer therapy. siRNAs were synthesized to interfere with ZFPM2-AS1 expression. qRT-PCR assay was utilized to verify the interference effect. Figure 3A showed that ZFPM2-AS1 expression was significantly decreased after transfection of siRNA, indicating successful interference. After thyroid cancer SW579 and 8505C cells transfected with si-ZFPM2-AS1, CCK-8 was presented to monitor the cell growth and to draw the cell growth curve. The results showed that the cell viability was significantly decreased following inhibiting ZFPM2-AS1 expression (Figure 3B). SW579 and 8505C cells transfected with si-ZFPM2-AS1 were suspended into single cells and seeded on plates, and cultured continuously for 14 days. After formaldehyde fixation and crystal violet staining, the number of clones on the plate was counted to exhibit the cloning capacity of the cells. Figure 3C showed that the number of cell clones was significantly reduced after down-regulating FPM2-AS1. EdU assay was utilized to test cell apoptosis. Apoptotic rate was significantly increased in cells transfected with si-ZFPM2-AS1 (Figure 3D, E). Furthermore, we examined the expression of two apoptosis-related proteins (Caspase 3/9). The data suggested that ZFPM2-AS1 knockdown significantly increased the expression of above proteins (Figure 3F). These results demonstrated that ZFPM2-AS1 knockdown could induce apoptosis of thyroid cancer cells.

### ZFPM2-AS1 knockdown inhibits migration and invasion of thyroid cancer cells

**Figure 4:**
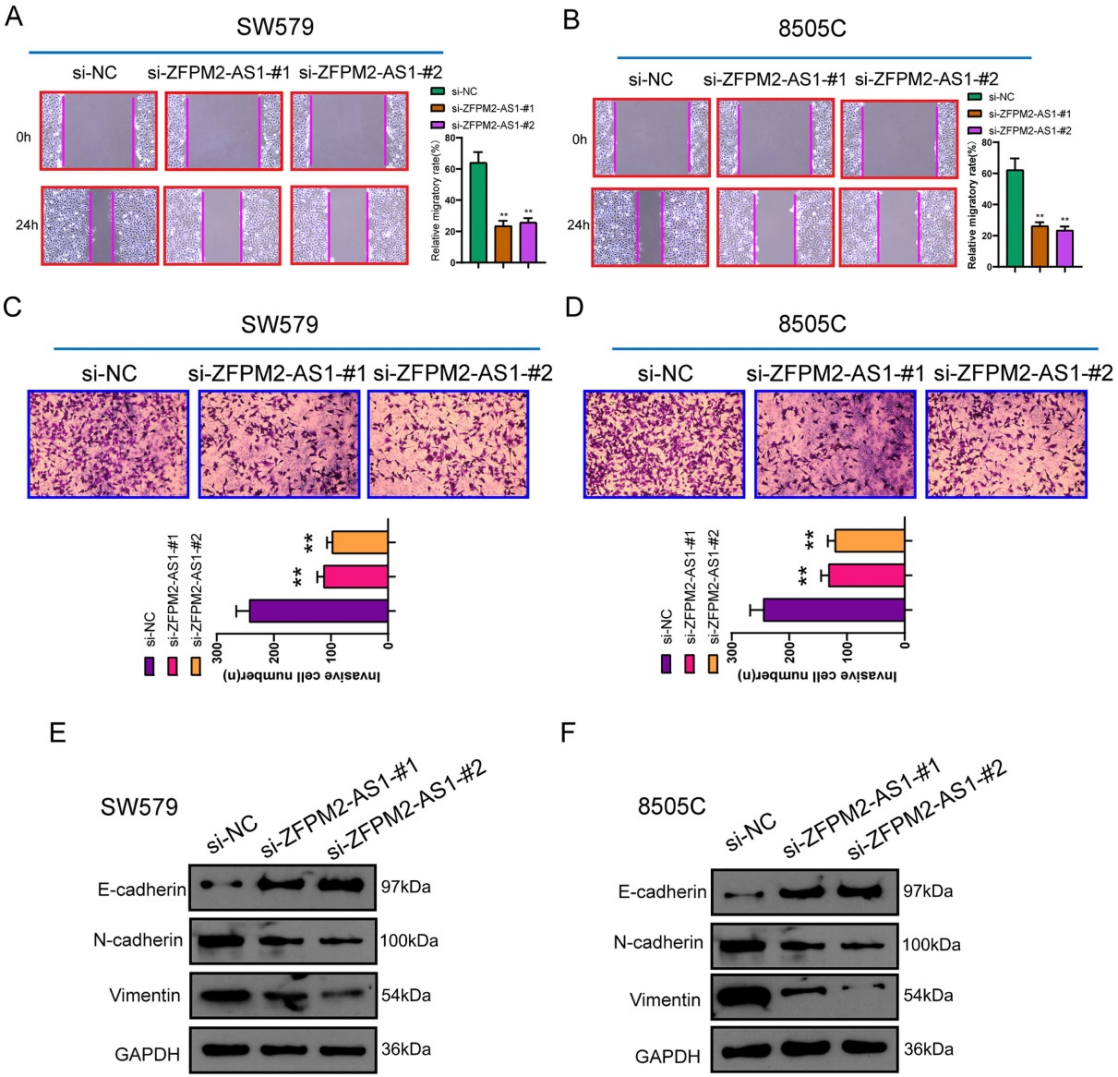
** ZFPM2-AS1 knockdown inhibits migrated and invasive capacities of thyroid cancer cells. (A, B)** Lower migratory rate was detected in SW579 and 8505C cells with si-ZFPM2-AS1. Magnification: 200×. **(C, D)** The number of cells that penetrated Matrigel to reach the lower layer of the chamber was reduced for cells with si-ZFPM2-AS1. Magnification: 200×. **(E, F)** Western blot was utilized to examine EMT-related markers (E-cadherin, N-cadherin and Vimentin) in SW579 and 8505C cells interfered with si-ZFPM2-AS1. **p<0.01.

After transfection of si-ZFPM2-AS1 in thyroid cancer SW579 and 8505C cells, wound healing and Transwell assays were performed to detect changes in migrated and invasive abilities. These results showed that after down-regulating ZFPM2-AS1, migratory rate was distinctly decreased (Figure 4A, B). Furthermore, the number of cells that penetrated the matrigel and migrated to the lower layer of the chamber was significantly reduced (Figure 4C, D). Western blot was used to detect changes in epithelial-mesenchymal transition (EMT)-related proteins. The data demonstrated that epithelial marker E-cadherin expression was raised, while mesenchyme marker (N-cadherin and Vimentin) expression was weakened, suggesting that silencing ZFPM2-AS1 inhibited EMT process (Figure 4E, F). This indicated that ZFPM2-AS1 on regulating thyroid cancer cell invasion and migration may be partly related to EMT process.

### ZFPM2-AS1 serves as a sponge for miR-515-5p in thyroid cancer cells

**Figure 5:**
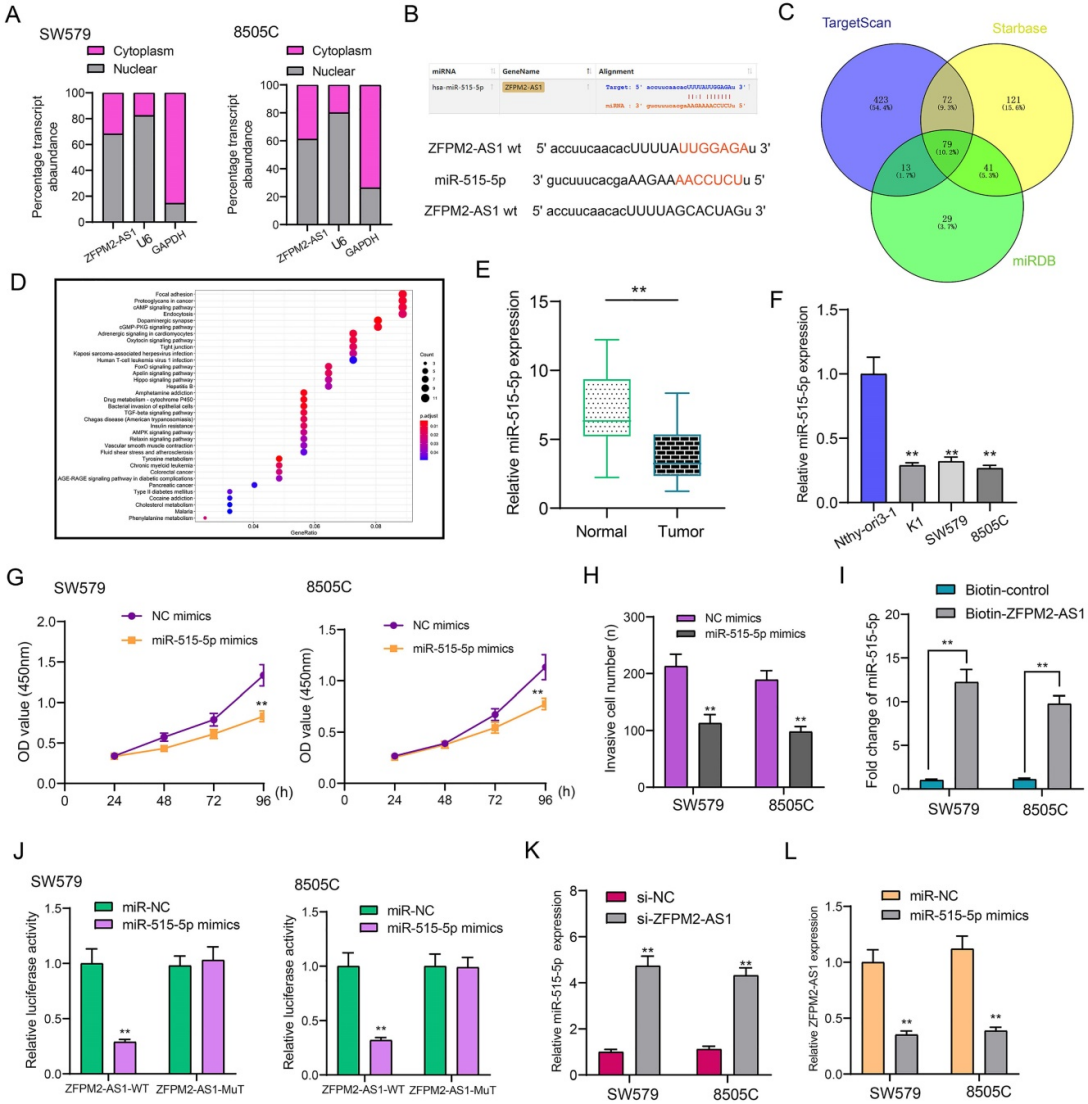
** ZFPM2-AS1 may sponge miR-515-5p in thyroid cancer cells. (A)** Subcellular localization of ZFPM2-AS1. **(B)** Putative binding sites between ZFPM2-AS1 and miR-515-5p. **(C)** Potential targeted mRNAs of miR-515-5p. **(D)** KEGG enrichment analyses of target mRNAs. **(E, F)** qRT-PCR was utilized to examine miR-515-5p expression in thyroid cancer tissues as well as cells. **(G, H)** Cell viability and invasion were assessed in cells with miR-515-5p mimics using CCK-8 and Transwell. **(I, J)** Pull-down and luciferase reporter assays confirmed the direct binding between ZFPM2-AS1 and miR-515-5p. **(K, L)** qRT-PCR examined the expressions of miR-515-5p or ZFPM2-AS1 in cells with si-ZFPM2-AS1 or miR-515-5p mimics. **p<0.01.

Subcellular localization confirmed that ZFPM2-AS1 was distributed in the nucleus and cytoplasm of thyroid cancer cells (Figure 5A). Through the starbase database (http://starbase.sysu.edu.cn/), ZFPM2-AS1 may specifically bind to miR-515-5p (Figure 5B). Through the TargetScan (http://www.targetscan.org/vert_72/), Starbase and miRDB (http://mirdb.org/) databases, a total of 79 potential target mRNAs of miR-515-5p were predicted in Figure 5C. KEGG enrichment analysis revealed that target mRNAs could mediate various pathways like focal adhesion, proteoglycans in cancer and cAMP signaling pathway (Figure 5D). In line with qRT-PCR results, low miR-515-5p expression was tested in thyroid cancer tissues in comparison to normal tissues (Figure 5E). Also, its down-regulation was found in thyroid cancer cells (Figure 5F). After transfection with miR-515-5p mimics, cell viability (Figure 5G) and invasion (Figure 5H) were both decreased in SW579 and 8505C cells. Pull-down assay results showed that ZFPM2-AS1 could pull down miR-515-5p (Figure 5I). Wild-type (wt) and mutant (mut) ZFPM2-AS1 luciferase reporter vectors were conducted. The co-transfection of ZFPM2-AS1-wt not ZFPM2-AS1-mut with miR-515-5p mimics greatly reduced the luciferase activity in SW579 and 8505C cells (Figure 5J). Furthermore, silencing ZFPM2-AS1 gained miR-515-5p expression (Figure 5K), while miR-515-5p mimics decreased ZFPM2-AS1 expression (Figure 5L). Thus, ZFPM2-AS1 may serve as a sponge for miR-515-5p in thyroid cancer cells.

### TUSC3 is a target mRNA of miR-515-5p in thyroid cancer

**Figure 6:**
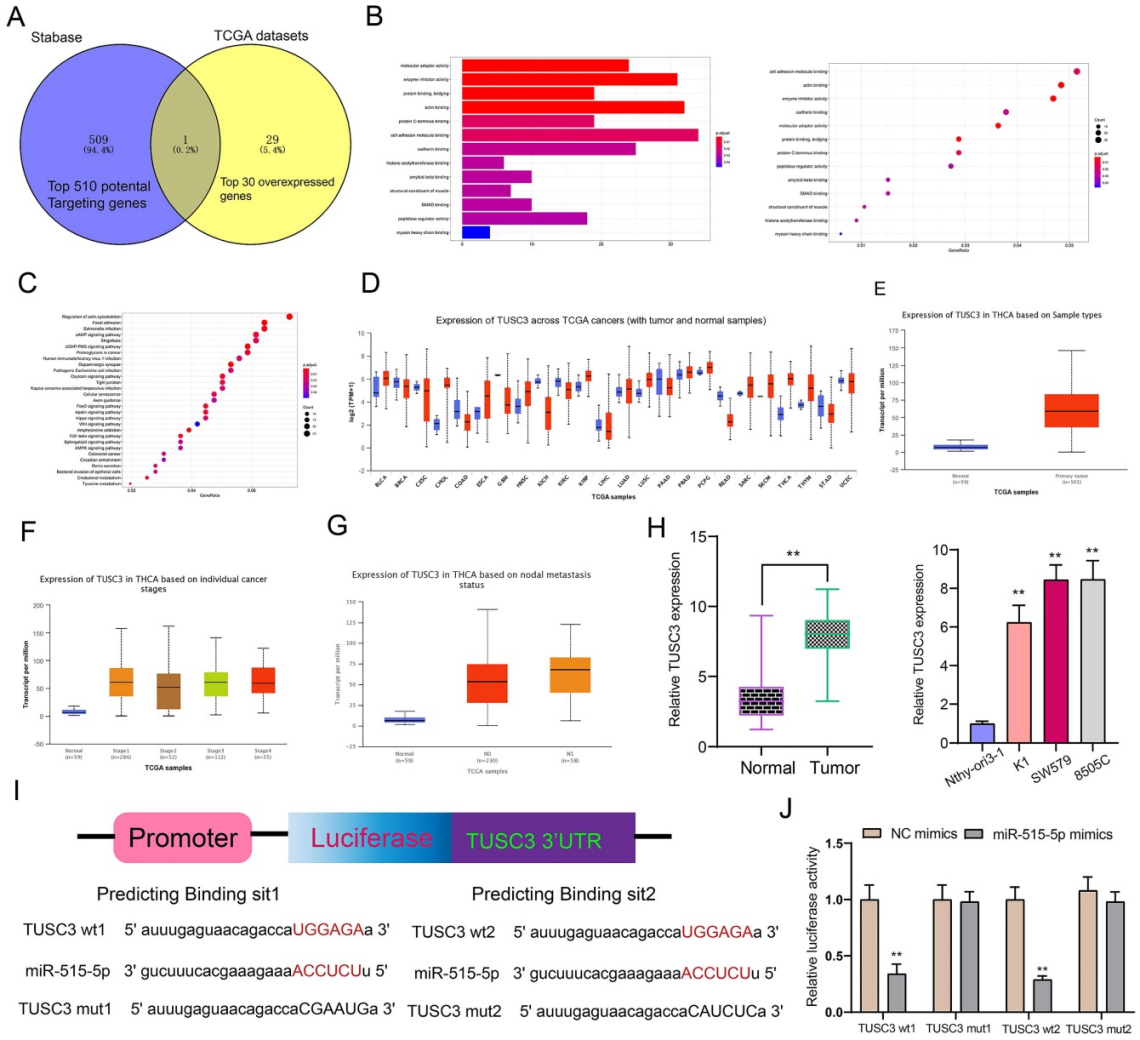
** TUSC3 is a targeted mRNA of miR-515-5p in thyroid cancer. (A)** Overlapping target mRNAs of miR-515-5p and overexpressed mRNAs in thyroid cancer. **(B, C)** Molecular functions and KEGG pathway analysis of target mRNAs. **(D)** Expression patterns of TUSC3 across different cancers using TCGA database. **(E)** Differences in expression patterns of TUSC3 in thyroid cancer and normal tissues. **(F)** Differences in expression patterns of TUSC3 in different clinical stages. **(G)** Differences in expression patterns of TUSC3 in nodal non-metastasis and metastasis status. **(H)** qRT-PCR was utilized to verify TUSC3 expression in thyroid cancer tissues as well as cells. **(I, J)** Dual luciferase report validated that miR-515-5p bound to the two binding sites in the 3'UTR region of TUSC3. **p<0.01.

Using the Stabase database, 510 underlying targeted mRNAs of miR-515-5p were predicted. Furthermore, the top 30 overexpressed mRNAs in thyroid cancer were screened out using TCGA database. After comprehensive analysis, TUSC3 was identified for further analysis (Figure 6A). Also, we explored potential molecular functions (Figure 6B) and KEGG pathways for target mRNAs (Figure 6C). The results showed that they were mainly involved in various key molecular functions and signaling pathways, indicating that these mRNAs could be associated with the development of thyroid cancer. On the basis of TCGA database, we found that TUSC3 exhibited different expression patterns in different cancers (Figure 6D). Intriguingly, it was over-expressed in thyroid cancer than normal tissues (Figure 6E). Moreover, high TUSC3 expression was detected in different clinical stages in comparison to normal samples (Figure 6F). Its expression was both distinctly higher in non-lymph node metastasis or metastasis samples than normal samples (Figure 6G). qRT-PCR was presented to validate its expression in thyroid cancer. Consistent with bioinformatics results, its up-regulation was discovered in thyroid cancer and cells (Figure 6H). The dual luciferase report confirmed that miR-515-5p bound to two binding sites in the 3'UTR region of TUSC3 (Figure 6I). After co-transfection of miR-515-5p mimics and TUSC3-wt1/2, luciferase activity was significantly decreased (Figure 6J). Collectively, miR-515-5p could mediate TUSC3 expression in thyroid cancer.

### miR-515-5p may suppress proliferation and invasion for thyroid cancer cells by inhibiting TUSC3 expression

**Figure 7:**
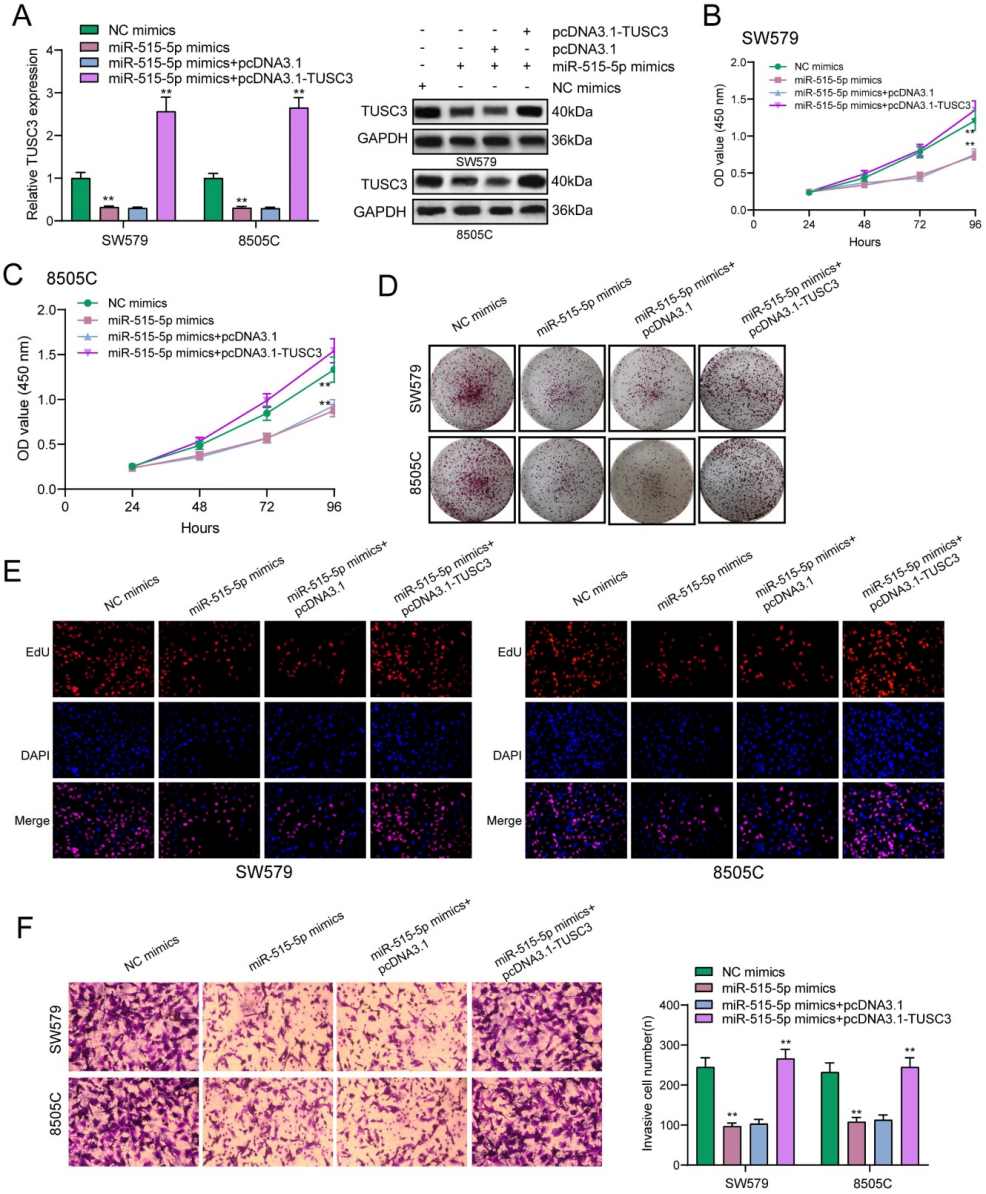
** miR-515-5p may suppress proliferation and invasion for thyroid cancer cells by inhibiting TUSC3 expression. (A)** Western blot detecting the expression of TUSC3 protein after transfection with miR-515-5p mimics and / or pcDNA3.1-TUSC3 in SW579 and 8505C cells. **(B, C)** CCK-8 assay was presented to examine the cell viability of SW579 and 8505C cells following transfection with miR-515-5p mimics and / or pcDNA3.1-TUSC3. **(D)** Clone formation experiment of transfected two thyroid cancer cells. **(E)** Edu assay results for transfected two thyroid cancer cells. **(F)** Transwell assay results showing the invasive ability of SW579 and 8505C cells transfected with miR-515-5p mimics and / or pcDNA3.1-TUSC3. **p<0.01.

The regulatory mechanisms of miR-515-5p and TUSC3 were further explored in this study. In Figure 7A, miR-515-5p mimics distinctly suppressed the expression of TUSC3 protein in thyroid cancer cells, which was ameliorated by TUSC3 overexpression according to western blot results. CCK-8 results demonstrated that TUSC3 overexpression significantly reversed the reduction in cell viability induced by miR-515-5p in SW579 (Figure 7B) and 8505C cells (Figure 7C). Furthermore, miR-515-5p-induced reduction in clonal formation (Figure 7D) and proliferation (Figure 7E) was improved after transfection with TUSC3 overexpression in thyroid cancer cells. In Figure 7F, TUSC3 overexpression enhanced the invasive ability induced by miR-515-5p mimics. Collectively, miR-515-5p may suppress proliferation and invasion for thyroid cancer cells by inhibition of TUSC3 expression.

### ZFPM2-AS1 mediates TUSC3 expression via miR-515-5p in thyroid cancer cells

**Figure 8:**
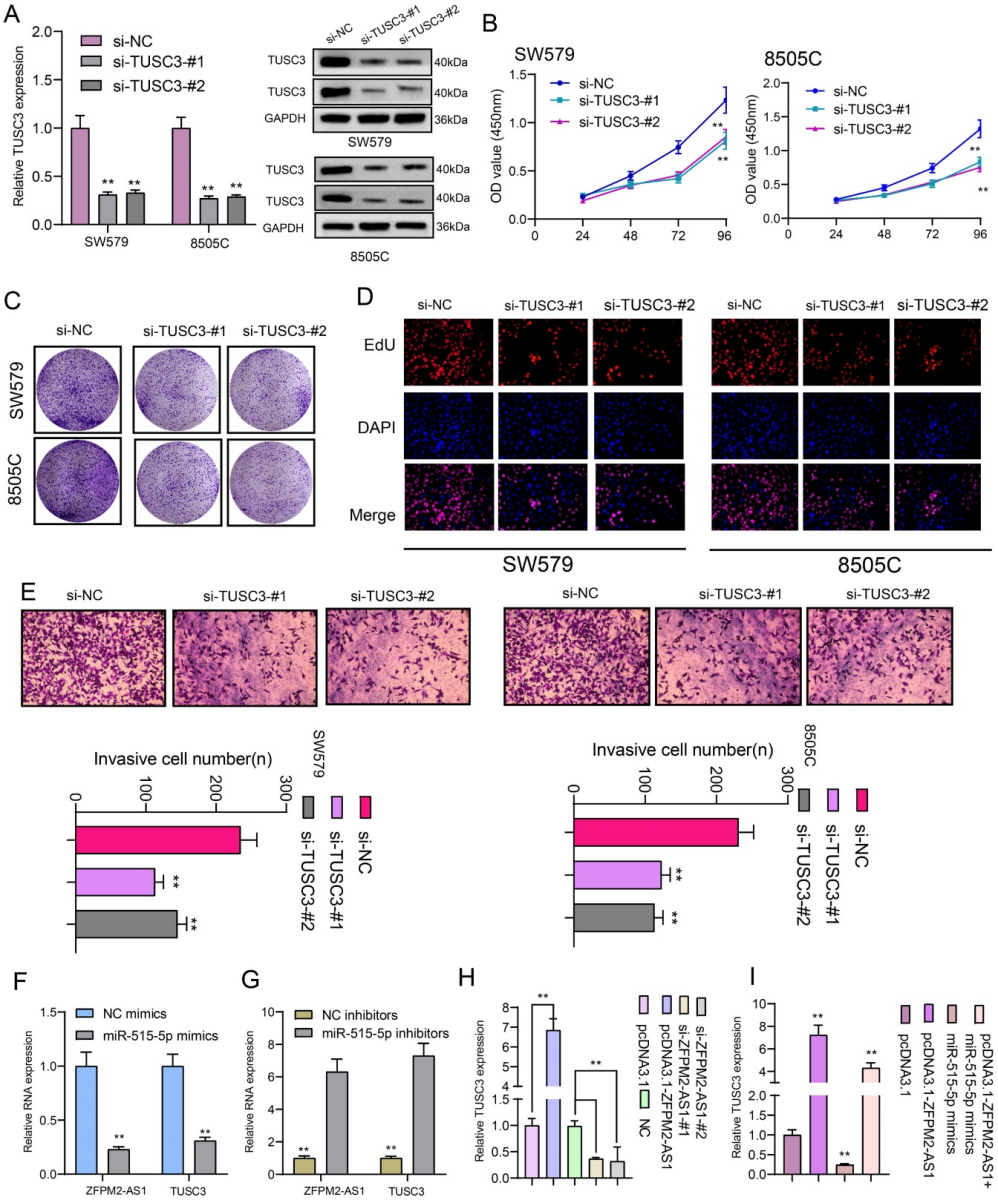
** ZFPM2-AS1 mediates TUSC3 expression via miR-515-5p in thyroid cancer cells. (A)** Transfection efficiencies of si-TUSC3 in SW579 and 8505C cells were verified using western blot. **(B-D)** Cell proliferation was detected in cells transfected with si-TUSC3 using CCK-8, clone formation and EdU assays. **(E)** Invasive capacity was assessed when transfected with si-TUSC3. **(F, G)** ZFPM2-AS1 and TUSC3 expression was validated after transfection with miR-515-5p mimics / inhibitors using qRT-PCR. **(H)** TUSC3 expression was determined when transfection with pcDNA3.1-ZFPM2-AS1 or si-ZFPM2-AS1 via qRT-PCR. **(I)** qRT-PCR was utilized to assess TUSC3 expression in cells transfected with pcDNA3.1-ZFPM2-AS1 and / or miR-515-5p mimics. **p<0.01.

**Figure 9:**
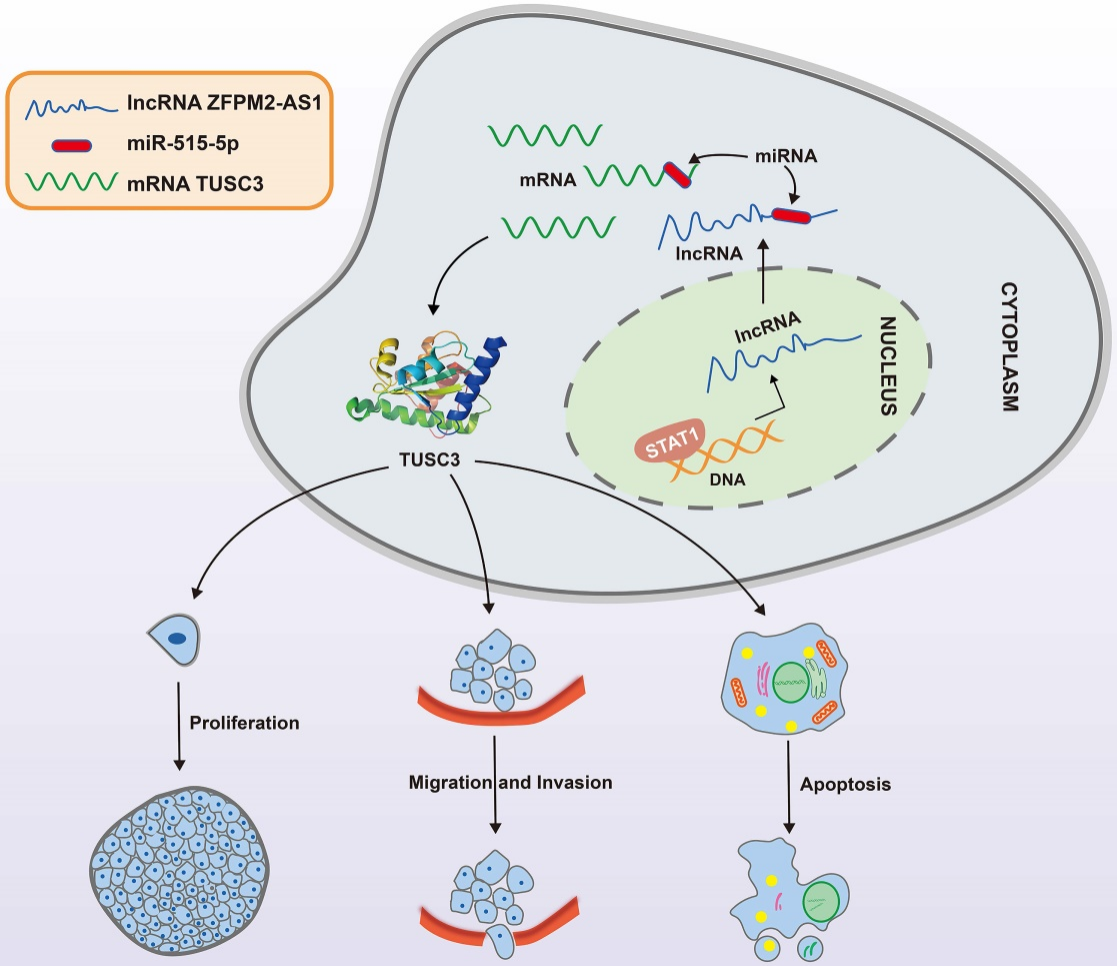
A mechanism diagram of STAT1/ZFPM2-AS1/miR-515-5p/TUSC3 axis in thyroid cancer.

We further observed si-TUSC3 on the biological behaviors of thyroid cancer cells. Two siRNAs were employed to silence TUSC3 expression in SW579 and 8505C cells. Western blot results confirmed that TUSC3 expression was successfully silenced (Figure 8A). Cell proliferation was conspicuously decreased after its knockdown in line with CCK-8, clone formation and EdU assays (Figure 8B-D). Furthermore, silencing TUSC3 distinctly inhibited cell invasive capacity of SW579 and 8505C cells (Figure 8E). Following transfection with miR-515-5p mimics, ZFPM2-AS1 and TUSC3 expression was both distinctly decreased (Figure 8F). Opposite results were detected in cells transfected with miR-515-5p inhibitors (Figure 8G). Moreover, TUSC3 expression was significantly increased by ZFPM2-AS1 overexpression, which was decreased by ZFPM2-AS1 knockdown (Figure 8H). miR-515-5p distinctly inhibited TUSC3 expression in thyroid cancer cells, which was reversed after co-transfection of pcDNA3.1-ZFPM2-AS1 (Figure 8I). These data suggested that ZFPM2-AS1 could mediate TUSC3 expression via miR-515-5p. Figure 9 depicted the regulatory mechanism of ZFPM2-AS1/miR-515-5p/TUSC3 pathway in thyroid cancer.

## Discussion

We firstly explored ZFPM2-AS1 expression in thyroid cancer. Its expression was up-regulated in thyroid cancer, furthermore, its high expression was positively associated with clinical stage, indicating that ZFPM2-AS1 might participate in promoting thyroid cancer progression. Studies have found that high ZFPM2-AS1 expression is an adverse prognostic factor of several cancers, such as gastric cancer 7, renal cell carcinoma 8 and lung adenocarcinoma 11. Abnormal expression of ZFPM2-AS1 affected the biological behavior of thyroid cancer cells. Mechanistically, ZFPM2-AS1 transcriptionally regulated by STAT1, could bind miR-515-5p through competitively competing with TUSC3 in thyroid cancer cells.

At present, the effects of ZFPM2-AS1 on the behavior of thyroid cancer cells have not been reported yet. Therefore, we employed siRNAs to interfere with ZFPM2-AS1 expression in thyroid cancer cells. Then we probed into the effects of tumor cell proliferation, apoptosis, migration as well as invasion after lowering ZFPM2-AS1 through a series of experiments. Through CCK8 and cloning formation experiments, it was found that the proliferation ability of tumor cells was significantly decreased after SW579 and 8505C cells transfected with si-ZFPM2-AS1. EdU results showed that apoptosis was distinctly increased for thyroid cancer cells with ZFPM2-AS1 knockdown. At the same time, ELISA results showed that the expression of apoptosis-related proteins was increased. Early thyroid cancer has low invasiveness and an optimistic prognosis after treatment. At present, most patients can achieve well clinical outcomes through surgery, but for some patients with high invasiveness and strong metastatic ability, the treatment effect is unsatisfactory 2. Therefore, finding key molecules and regulatory pathways that regulate the invasion and metastasis processes is the focus of thyroid cancer research, which will help to diagnose some patients with poor prognosis as early as possible in clinical practice, thereby providing more appropriate treatment options. Our experiments demonstrated that ZFPM2-AS1 knockdown could inhibit the migrated and invaded abilities of thyroid cancer cells. The invasion and migration of tumor cells are inseparable from EMT process 14. EMT is the core initial step of tumor invasion and metastasis 15. Primary tumor epithelial cells usually lose their cell polarity, and the tight connection with the basement membrane disappears, and finally exhibits mesenchymal phenotype 16. On this basis, the capacities of tumor cells to move, invade, migrate, and degrade extracellular matrix are significantly enhanced. After the occurrence of EMT, tumor cells leave the basement membrane, cross the gap between vascular endothelial cells, enter the blood circulation, and eventually transfer to distant organs. During this process, the expression of specific epithelial cell adhesion molecule E-cadherin is weakened, while interstitial marker (like Vimentin and N-cadherin) expression is raised. The reduction or deletion of E-cadherin expression is a primary link for tumor cell invasion and migration. Herein, western blot results verified that si-ZFPM2-AS1 remarkably induced E-cadherin expression and receded Vimentin and N-cadherin expression, thereby inactivating EMT process. The above experiments suggested that ZFPM2-AS1 can affect the biological behaviors of thyroid cancer cells *in vitro*.

As previous studies, multiple transcription factors are up-regulated in thyroid cancer 17-19. Among them, several transcription factors can induce the up-regulation of lncRNAs, such as FOXO1 20 and SP1 21. In this study, our data confirmed that STAT1 could direct bind to the promoter region of ZFPM2-AS1, thereby up-regulating ZFPM2-AS1 expression. LncRNA functions as a sponge of miRNA in the cytoplasm 22. ZFPM2-AS1 was expressed in nucleus and cytoplasm of thyroid cancer cells. The direct association between ZFPM2-AS1 and miR-515-5p was validated using RNA pull-down and luciferase report. This miRNA was lowly expressed in thyroid cancer, partly due to the inhibition of ZFPM2-AS1. Its up-regulation suppressed thyroid cancer cell viability. Previously, miR-515-5p can act as a tumor suppressor like breast cancer 23, lung cancer 24 and prostate cancer 25. We found that TUSC3 was a target of miR-515-5p, which was over-expressed in thyroid cancer. Its up-regulation has been detected in several cancers such as colorectal cancer 26, glioblastoma 27, clear cell renal cell carcinoma 28, lung cancer 29. Dual luciferase report confirmed that miR-515-5p bound to the two binding sites in the 3'UTR region of TUSC3. Previously, it was targeted by several miRNAs like miR-181b-5p 30, miR-181a-5p 31, miR-30e-5p 31. TUSC3 knockdown prominently suppressed proliferative and invasive capacities of thyroid cancer cells. Thus, ZFPM2-AS1 bound to miR-515-5p via competitive competition with TUSC3 in thyroid cancer cells.

## Conclusion

Taken together, ZFPM2-AS1 acts as an oncogene in thyroid cancer. Its up-regulation is related to tumor progression. In the nucleus, it is transcriptionally mediated by STAT1. In the cytoplasm, ZFPM2-AS1 weakens the inhibitory effect of miR-515-5p on TUSC3. These findings propose a strategy for ZFPM2-AS1 as an underlying marker for thyroid cancer.

## Abbreviations

lncRNA: long non-coding RNA
ZFPM2-AS1: ZFPM2 Antisense RNA 1
qRT-PCR: quantitative real-time PCR
CHIP: Chromatin immunoprecipitation
CCK-8: Cell counting kit-8
EdU: 5-Ethynyl-2'-deoxyuridine
EMT: epithelial-mesenchymal transition
ELISA: enzyme-linked immunosorbent assay
